# Exploring the prevalence and risk factors of adolescent mental health issues in the COVID and post-COVID era in the U.K.: A systematic review

**DOI:** 10.17179/excli2025-8325

**Published:** 2025-03-27

**Authors:** Kelvin Kanayo Nwabueze, Nnaemeka Akubue, Ademola Onakoya, Stella Chidinma Okolieze, Ikponmwosa J. Otaniyen-Igbinoba, Chisom Chukwunonye, Chinelo Grace Okengwu, Temiloluwa Ige, Oluwaseyi Joy Alao, Kelechi Nelson Adindu

**Affiliations:** 1 Acute Medicine Department, Watford General Hospital, West Hertfordshire NHS Trust, U.K.; 2 School of Allied & Public Health, University of Chester, Chester, U.K.; 3 Department of Primary Care, Old Catton Medical Practice (NHS), Norwich, U.K.; 4 Department of Biomedical Science, Edinburgh Napier University, Edinburgh, Scotland, U.K.; 5 Department of Family Medicine, Lily Hospitals Limited, Warri, Nigeria; 6 Department of General Medicine, Betsi Cadwaladr University Health Board, Wales, U.K.; 7 Department of General Medicine, The Royal Shrewsbury and Telford (NHS), Shrewsbury, U.K.; 8 Department of Psychiatry, Herefordshire and Worcestershire Health and Care NHS Trust, Worcester, U.K.

**Keywords:** adolescents, United Kingdom, mental health, prevalence, risk factors, COVID-19

## Abstract

Adolescence is a developmental phase largely characterized by rapid biological and non-biological transformations, with a heightened susceptibility to social and environmental influences. Hence, adolescents are particularly vulnerable to external stressors, underscoring the need to safeguard their well-being and prioritize mental health interventions. The coronavirus disease (COVID-19) pandemic caused a global crisis with profound societal disruptions, and led to lasting impact on global public health, disproportionately affecting vulnerable populations, including adolescents. In view of the unique developmental challenges faced by adolescents, it is imperative to assess the growing burden of mental health issues exacerbated by the pandemic. This review synthesizes existing evidence on the emerging mental health challenges faced by adolescents in the United Kingdom (UK) as exacerbated by the COVID-19 pandemic. A systematic literature search was conducted using PubMed, ScienceDirect, MEDLINE, and SpringerNature databases, resulting in the selection of ten high-quality studies. A thematic analysis of the collected data revealed that depression and anxiety were the most frequently reported mental health conditions among adolescents. These conditions were particularly prevalent among adolescents who were from low-income households, those with pre-existing mental health disorders, adolescents experiencing household conflicts, females, and those who provided self-reported data. Several key risk factors were identified, including family and peer relationships, academic pressures such as examinations and grades, financial constraints within households, and the corruptive influence of social media. The findings underscore the urgency of targeted mental health interventions tailored to the specific needs of adolescents in the U.K. By addressing the identified risk factors, mental health professionals, policymakers, and educators can develop more effective strategies to mitigate the psychological impact of the pandemic on this vulnerable population. This study contributes to the evolving body of literature and emphasizes the need for evidence-based policies to foster overall well-being and resilience in adolescents navigating post-pandemic challenges.

## Background

The impact of the 2020 coronavirus disease (COVID-19) pandemic has been widely documented across all demographics, with particularly pronounced effects on vulnerable populations, including children and adolescents (Chavira et al., 2022[10]; Deng et al., 2023[15]; Hassan et al., 2023[20]). This increased vulnerability can be attributed to the extensive disruptions to societal activities and the restrictive interventions implemented to mitigate the spread of the virus, all of which significantly altered the activities of daily living and posed substantial threats to psychological well-being (Deng et al., 2023[15]). Consequently, the pandemic-induced societal changes have had profound implications for adolescent development and mental health.

The World Health Organization (WHO) defines adolescence as the period between 10 and 19 years of age (WHO, 2025[43]). In the United Kingdom (U.K.), individuals within this age group constitute approximately 12 % of the total population (AYPH, 2024[5]). Adolescence is a phase marked by heightened curiosity and a strong susceptibility to environmental and peer influences, thus increasing the likelihood of psychological distress under adverse conditions (Knowles et al., 2022[24]). Even before the pandemic, adolescent mental health was a growing concern, and the COVID-19 crisis exacerbated existing challenges (Angelina et al., 2021[3]; Chavira et al., 2022[10]). The pandemic not only imposed physical restrictions but also introduced profound emotional distress, particularly due to bereavement following the loss of loved ones, further compromising the psychological resilience of affected adolescents (Chavira et al., 2022[10]; Deng et al., 2023[15]). Moreover, mental health conditions often remain undetected due to their insidious nature, as clinical manifestations of psychological distress tend to be less overt than physical health symptoms (Deng et al., 2023[15]; Hassan et al., 2023[20]).

The pandemic's enduring influence continues to shape various facets of global society. Deng et al. (2023[15]) projected the long-term repercussions of this crisis on mental health, particularly due to increased screen time, reduced physical activity, and diminished social engagement-factors that have been widely acknowledged as contributors to psychological distress (Angelina et al., 2021[3]; Deng et al., 2023[15]). For example, the transition from in-person education to remote virtual learning, significantly restricted students' social interactions, exacerbating feelings of isolation and emotional strain (Angelina et al., 2021[3]; Hassan et al., 2023[20]). Additionally, the economic downturn resulting from pandemic-associated job losses led to substantial reductions in household income, forcing lifestyle adjustments that further intensified socio-psychological stressors for adolescents (Angelina et al., 2021[3]).

There is strong evidence supporting the assertion that the pandemic has had a profound impact on adolescent mental health (Angelina et al., 2021[3]). Given the importance of early detection in health promotion and the prevention of severe psychological complications, there is a dire need to investigate emerging trends in adolescent mental health challenges post-pandemic (Deng et al., 2023[15]). Interestingly, a significant proportion of adolescents who experience mental health challenges remain undiagnosed (Nebhinani and Jain, 2019[30]), increasing their susceptibility to self-destructive behaviors, including substance abuse and suicide (Deng et al., 2023[15]). While numerous studies have explored the psychological toll of the pandemic on adolescents in different regions (Angelina et al., 2021[3]; Chavira et al., 2022[10]), much of the existing literature, such as meta-analyses, tends to focus on global prevalence rates (Deng et al., 2023[15]). Furthermore, studies linking adolescent mental health issues to substance abuse, such as the one conducted by Temple et al. (2022[38]), have primarily been centered on the United States. However, researchers argue that studies with a more localized focus are essential, as global analyses may obscure regional trends and demographic-specific risk factors (Knowles et al., 2022[24]).

In view of these considerations, this study aims to examine the prevalence of mental health challenges among adolescents in the U.K. in the wake of the COVID-19 pandemic. Additionally, it seeks to identify key risk factors contributing to these emerging trends, thereby providing relevant region-specific insights to inform targeted interventions.

## Method

The research focus was framed using the SPIDER framework, representing Sample, Phenomenon of Interest, Design, Evaluation, and Research type, which is recommended in coining research questions during systematic reviews (Methley et al., 2014[28]). It is useful in non-clinical health research and aids in developing the eligibility criteria used in performing the literature search (Amir-Behghadami, 2024[2]). While other commonly used frameworks exist, the SPIDER tool is more suited for qualitative synthesis of data than PICO (Population, Intervention, Comparator, Outcome) and more specific in usage than PEO (Population, Exposure, and Outcome) (Cooke et al., 2012[14]). It was hence used as follows:

S - Adolescent in the U.K.

PI - Mental health issues in the COVID and post-COVID era

D - Qualitative, quantitative, mixed

E - Prevalence, risk factors

R - Quantitative

### Eligibility criteria

This was developed from the research framework and produced the set of rules to be considered when selecting literature for the review. The inclusion criteria are therefore given as:

- Studies published in peer-reviewed journals
- Studies conducted in the United Kingdom
- Primary research
- Studies focused on adolescents or young adults
- Studies addressing mental, psychological, or psychosocial health
- Studies published between 2020 and 2025.
- The exclusion criteria were given as:
- Unavailable texts
- Studies that are reported in languages other than English
- Studies with low methodological quality.

### Literature search

The search was conducted with the use of search terms formed from the keywords associated with the SPIDER research framework as applied in this review. In accordance with standards (Grewal et al., 2016[18]), Boolean operators were used to link these search terms and their synonyms to form a string given as: “(Adolescent OR adolescence OR young adult) AND (UK OR United Kingdom) AND (mental health OR psychological) AND (COVID OR post-COVID OR coronavirus pandemic) AND (issues Or problem OR challenge) AND prevalence AND risk factors”.

The selected databases for this search were PubMed, ScienceDirect, MEDLINE, and SpringerNature. Due to the restrictions faced in the use of ScienceDirect, the search string was modified while using the database, giving as “(Adolescent/adolescence) AND (UK/United Kingdom) AND (mental health/ psychological) AND (COVID) AND (issues/problem/challenge) AND prevalence AND risk factors”.

Following this process, quality assessment was conducted using the Critical Appraisal Skills Programme (CASP) checklist for the qualitative and cohort studies as illustrated by Table 1(Tab. 1) (References in Table 1: Hu and Qian, 2021[21]; Karamanos et al., 2022[23]; Knowles et al., 2022[24]; Montero-Marin et al., 2023[29]; Wright et al., 2020[44], 2024[45]) and Table 2(Tab. 2) (References in Table 2: Anto et al., 2023[4]; Lee and Wong, 2024[25]; Spencer et al., 2022[36]), while Joanna Briggs Institute (JBI) assessment tool which is highlighted by Table 3(Tab. 3) (Reference in Table 3: Mansfield et al., 2021[27]), was used to evaluate the cross-sectional studies.

### Data analysis

The data obtained from all included studies were analyzed thematically using the Braun and Clarke's model (2023[8]). It involves 6 steps as further expounded by Braun et al. (2016[9]). The first step is to be familiarized with the collected data, which in this case involves repeated study of each paper for optimal assimilation. The second step involves the generating of codes using keywords and recurring phrases found in the individual studies during the data familiarization. The next three steps are to develop, refine, and name themes. These themes are formed as a merged concept from related codes, which is refined to give a flow of discussion and named as the topics of this discussion, recognized finally as themes (Clarke and Braun, 2017[12]). The last step is the overall development and presentation of the analysis results to form the findings and interpretation (Braun et al., 2016[9]). 

## Results

**Figure 1:**
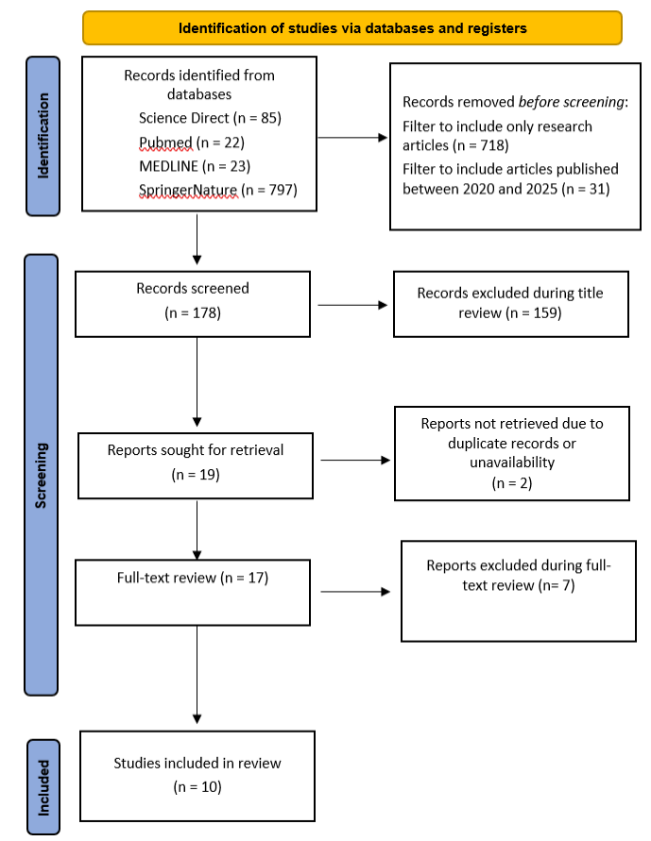
PRISMA flowchart illustrating the literature selection process (from Page et al., 2021) (PRISMA = Preferred Reporting Items for Systematic Reviews and Meta-Analyses)

The initial search yielded a total of 927 results, accounting for 85, 22, 23, and 797 results in ScienceDirect, PubMed, MEDLINE, and SpringerNature databases respectively. The date filter was then applied to return only studies published between 2020 and 2025, thereby eliminating 31 studies. Thereafter, filters were added to remove reports that were not research articles, open access, or full texts, further eliminating 718 reports. This led to the screening of 178 articles, after which 159 were removed during the review of titles as some were conducted as reviews, conducted outside the U.K., and had other demographic focus. The remaining articles were then subjected to full-text review, among which two articles were not retrieved due to duplicated records. In total, 17 articles were reviewed and seven were removed. The remaining 10 underwent and passed methodological appraisal. Hence, they were included in this review as shown in Figure 1(Fig. 1) (Reference in Figure 1: Page et al., 2021[31]).

## Findings

Following the literature search and selection process, six cohort studies, three qualitative studies, and one cross-sectional study were recruited for this review. Each of these studies showed a high methodological quality when the corresponding appraisal checklists were used to conduct the assessment. A summary of the basic characteristics is given in Table 4(Tab. 4) (References in Table 4: Anto et al., 2023[4]; Hu and Qian, 2021[21]; Karamanos et al., 2022[23]; Knowles et al., 2022[24]; Lee and Wong, 2024[25]; Mansfield et al., 2021[27]; Montero-Marin et al., 2023[29]; Spencer et al., 2022[36]; Wright et al., 2020[44], 2024[45]).

### Themes

**Figure 2:**
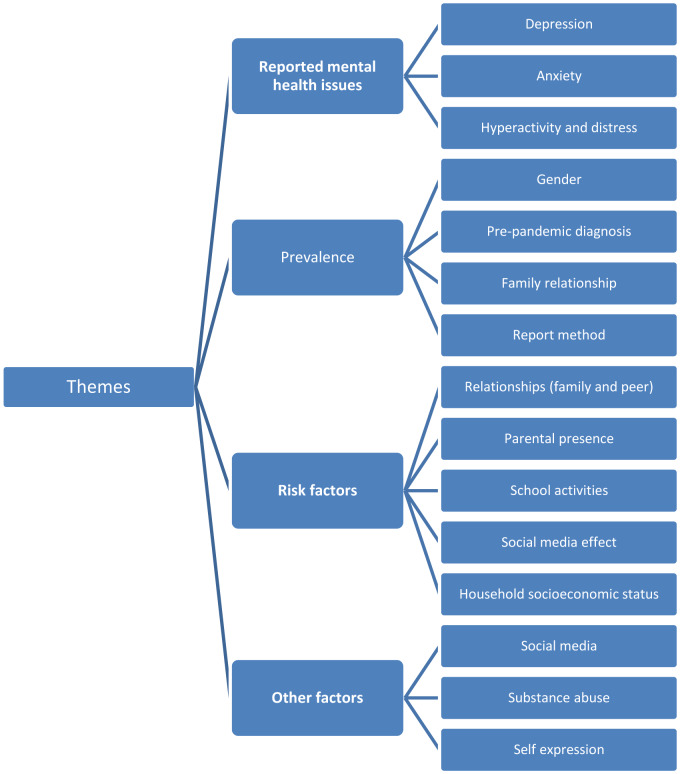
Generated themes

The analysis of data led to the derivation of four themes and they are illustrated graphically in Figure 2(Fig. 2).

#### Theme 1 - Reported mental health issues

In general, every included study provided evidence of the impact of the coronavirus pandemic on the mental health of adolescents in the U.K. Indeed, there is a general deterioration from the pandemic era (Hu and Qian, 2021[21]). For example, Montero-Marin et al. (2023[29]) compared two cohorts of pre-pandemic and pandemic era and, from the results, the second group showed a significant decrease in the quality of mental health, thereby proving the impact of COVID-19 on the adolescents. Also, Knowles et al. (2022[24]) conducted a study before, during, and after the pandemic. The pandemic period reported a significantly higher report of distress, estimated to increase by a factor of 0.43 on the average.

Furthermore, while some studies evaluated general mental health issues such (Karamanos et al., 2022[23]; Knowles et al., 2022[24]), the majority reported specific issues. The most occurring is depression, reported in six studies (Wright et al., 2020[44], 2024[45]; Mansfield et al., 2021[27]; Spencer et al., 2022[36]; Anto et al., 2023[4]; Montero-Marin et al., 2023[29]). Anxiety was the second most popularly recorded mental health issues as it was found in four studies (Mansfield et al., 2021[27]; Spencer et al., 2022[36]; Anto et al., 2023[4]; Montero-Marin et al., 2023[29]). Other conditions include hyperactivity, distress, and conduct problems (Hu and Qian, 2021[21]; Knowles et al., 2022[24]).

#### Theme 2 - Prevalence

Specific parameters were assessed in the cohort and cross-sectional studies which showed the extent to which the reported mental health issues occurred. Decreased social, emotional, and mental health was recorded in all adolescents, regardless of their demographic characteristics (Montero-Marin et al., 2023[29]). However, the resulting symptoms were more in females and adolescents with previous diagnoses of at least one psychological disorder (p<0.05) (Wright et al., 2020[44]; Mansfield et al., 2021[27]; Knowles et al., 2022[24]; Montero-Marin et al., 2023[29]). In one of these studies, girls reported higher incidence of depression but this started to reduce and slowly returned to the pre-pandemic state at the latter stages of follow-up, although this value remained higher than in boys (Wright et al., 2024[45]).

For those with pre-pandemic diagnosis, highly significant increase in hyperactivity was recorded, as well as peer relationship, conduct, and emotional problems (Hu and Qian, 2021[21]). In addition, a significant reduction in prosocial tendency was recorded. In terms of age, older adolescents had higher reports of depression (Wright et al., 2024[45]).

Again, those who reported to experience a closer family relationship during the pandemic also reported low psychological distress (p<0.05) when compared to the adolescents with reported incidents of domestic abuse, conflict, and/or violence (Knowles et al., 2022[24]; Montero-Marin et al., 2023[29]).

Also, data obtained from self-reported data collection tools showed a higher recorded prevalence of mental health issues than reports obtained from mothers of the target population (Wright et al., 2024[45]). During the comparison of the adolescents who spent the lockdown period at home and those who remained in school, the latter reported more symptoms of depression and anxiety than those who stayed at home (Mansfield et al., 2021[27]).

#### Theme 3 - Risk factors

An analysis of the risk factors associated with the reported mental health issues was also carried out in the included studies. Relationships stood out as a popularly reported factor that determined the state of the adolescent mental health. The influence of both family and peer relationships (p< 0.05) was reported in three quantitative studies and two qualitative studies (Mansfield et al., 2021[27]; Knowles et al., 2022[24]; Spencer et al., 2022[36]; Anto et al., 2023[4]; Montero-Marin et al., 2023[29]).

According to some adolescents, the lockdown itself was not an issue but the uncertainty of retaining physical relationships and the nature of the home environment (Montero-Marin et al., 2023[29]). People whose parents were essential workers statistically reported more depression symptoms (Mansfield et al., 2021[27]). The reports were also higher in homes where both parents are fully engaged in their occupations (Karamanos et al., 2022[23]).

Also, quoted texts from adolescents further illustrated this situation

*“the friends that I had at the time… we couldn't hang out in person. So we just then ended up not really texting and just drifted apart.”*

*“I didn't have a phone… I never got those numbers.”*

*“Friends were really a big help when it came to stuff like learning, the loneliness so like psychological impact…” (Lee and Wong, 2024*[25]*)*.

In contrast, some adolescents reported an Improved mental health due to the absence of adverse peer relations such as fighting and bullying (Hu and Qian, 2021[21]). This is in tandem with another study that highlighted positive student atmosphere in school as a significant risk factor but this association did not remain strong with time (Knowles et al., 2022[24]).

School grades was also widely referred to as an influencer of the instability of adolescents who had academic activities during the pandemic era (Knowles et al., 2022[24]). Those who were about to undertake national examinations showed greater symptoms of anxiety (Mansfield et al., 2021[27]). According to the result of an interview,

*“Near our exam time, most of my friends were quite sad and depressed because they felt like they were just sitting in the house and not really doing much and they were just consumed by all this exam stress, that they couldn't do anything” (Spencer et al., 2022*[36]*).*

A highly significant influence that continued to linger is the social media effect. The need to communicate virtually led to the impulse to always use mobile devices and led to stress of the fear of missing out.

*“I would not use my phone for even just 2 hours and I would feel like I was missing out on something. This would drive me crazy and add so much unnecessary stress” (Anto et al., 2023*[4]*).*

Its influence was reported on self-esteem due to increased comparison (Lee and Wong, 2024[25]). According to a quoted text,

*“When people post a lot of content about their lives whilst you're just at home, not doing anything special, you feel anxious in that you should really be doing something. Or that I am not doing enough” (Anto et al., 2023*[4]*).*

Spencer et al. (2022[36]) evaluated the impact of social media on adolescents and the most commonly reported mental health issues were depression and anxiety.

*“the whole like 'lockdown body challenge'… it affected a lot of things for me, like in terms of body image…” (Lee and Wong, 2024*[25]*).*

*“There were a few people who used to DM (direct message) me. They would talk about my appearance, the way I look and because I am quite self-conscious about that, they would use it to target me and harass me” (Anto et al., 2023*[4]*).*

Contrastively, a few reports showed a positive utilization of social media, which include reconnection with loved ones and achieving personal growth as a result. Directly quoting the respondents,

*“In terms of my mental well-being, it can help me stop loneliness and keep in touch with friends and family especially when I'm at university”*

*“I've met like some of my closest most dear friends through it”*

*“I can follow someone that has a healthy living account for example I find that seeing posts like that would encourage me to be better or to go out and go for a run or pick something healthy to do”(Anto et al., 2023*[4]*).*

Finally, household finances was another reported risk factor that led to emotional and mental instability due to the need for improvisation and survival (Knowles et al., 2022[24]).

*“Both of my parents lost their jobs due to COVID… that was difficult for them… That was a big hit because I have to fend for myself now” (Lee and Wong, 2024*[25]*).*

One of the studies concluded by stating statistically that the more factors present in the life of a typical UK adolescent, the greater the reported distress (Knowles et al., 2022[24])

#### Theme 4 - Other factors

Other factors reported in the studies include outcomes and positive influencers. The outcomes of the mental health issues include the newly formed habit of always engaging on social media, leading to an increased need for external validation in many adolescents (Anto et al., 2023[4]; Lee and Wong, 2024[25]). Substance abuse was also reported, which correlated with the male gender.

*“I had a bit of a drug problem… the vulnerability of being stuck indoors, we didn't have anything else to do” (Anto et al., 2023*[4]*).*

To explain this, other researchers posit that boys are more likely to have engaged in substance abuse as they are more likely to have witnessed and experienced physical assault or discipline while girls are more likely to be sexually assaulted, hence explaining their higher symptoms of psychological distress (Karamanos et al., 2022[23]). 

On the other hand, healthy habits were formed by come adolescents to keep their minds preoccupied and ultimately maintain their mental health.

*“Social connections and escapism were linked to help” (Anto et al., 2023*[4]*).*

Finally, the need for human interactions was buttressed as positivity was reported in those who had people to communicate and share their feelings with, thereby proving the significance of social relations and self expression in the psychological state of adolescents.

*“I haven't really spoken to anyone [about Covid], except like, you… this is the best time I've spoken of Covid” (Anto et al., 2023*[4]*).*

*”My form tutor knows more stuff, things, that's happening, because I was really close to him all the way through school. He knows my past, so I feel comfortable talking to him about it” (Spencer et al., 2022*[36]*).*

## Discussion

Adolescence is a critical developmental phase in which behavioral, psychological and social factors such as peer interactions and societal influences-play a pivotal role in shaping mental health outcomes (Knowles et al., 2022[24]). Biologically, the hormonal changes that characterize this developmental stage further contributes to the complexity of adolescent mental well-being (Knowles et al., 2022[24]). According to Bell et al. (2023[6]), 75 % of the reported mental health diseases in adults emerge in their adolescent stage. The intricate relationship between mental and physical health has been well-documented, with available evidence suggesting that psychological distress can manifest in physiological consequences. For instance, one of the reviewed studies reported weight gain among participants experiencing depression (Lee and Wong, 2024[16]). Additionally, Chavira et al. (2022[10]) demonstrated a strong correlation between depression, anxiety, and sleep deprivation, which further impacts on the adolescent's overall physical health and mental wellbeing. Furthermore, the heightened vulnerability of adolescents to self-harm and suicidal behaviors in response to psychological distress underscores the urgent need for robust mental health interventions tailored to this demographic (Panchal et al., 2023[32]; Trafford et al., 2023[39]). While these manifestations have been described as coping mechanisms in the affected individuals, they must be addressed from the root cause to improve the overall wellbeing of the adolescents (Akın and Sarrar, 2024[1]). A systematic review and meta-analysis of the effect of the pandemic on both children and adolescents statistically linked adolescence to the mental effects of COVID-19 (Panchal et al., 2023[32]). Hence, underscoring the significance of adolescence and the importance of safeguarding the mental health of the given population.

The findings of this review align with a growing body of literature documenting significant changes in adolescent mental health in the aftermath of the COVID-19 pandemic (Groarke et al., 2020[19]; Chavira et al., 2022[10]; Chepo et al., 2024[11]). Notably, a large-scale analysis of Twitter (now X) posts-comprising 300,000 entries-highlighted an increased prevalence of negative emotions among adolescents, with a substantial number of posts suggesting an urgent need for provision of psychological support and intervention (Chepo et al., 2024[11]). The robustness of this study, given its extensive dataset, further corroborates the widespread impact of the pandemic on adolescent mental health. Depression and anxiety have emerged as the most frequently reported conditions, largely attributed to the pervasive loneliness and social isolation resulting from lockdown measures (Groarke et al., 2020[19]; Bell et al., 2023[6]). This is equally reported in similar studies conducted in other countries such as Australia (Magson et al., 2021[26]; Bell et al., 2023[6]), China (Zhang et al., 2021[46]), the United Arab Emirates (UAE) (Ghader et al., 2024[17]), and the United States of America (USA) (Cohen et al., 2021[13]; Raney et al., 2024[34]). Indeed, the WHO estimated a 25 % increase in depression and anxiety in the global population, highlighting young people as one of the most affected demographics (WHO, 2022[42]).

Considering the prevalence patterns, this review's findings highlight a consistency with previous research, which identified females, adolescents from low-income households, and those from single-parent homes as being disproportionately affected by mental health challenges (Chavira et al., 2022[10]; Panchal et al., 2023[32]). These factors were reported in a study conducted on adolescents in Chinese secondary schools, recording parental relationships as one of the most influencing factors (Zhang et al., 2021[46]). Additionally, the method of data collection appears to influence reported prevalence rates, as studies indicate that self-reported assessments by adolescents often yield higher rates of mental health concerns compared to parental reports (Chavira et al., 2022[10]; Bell et al., 2023[6]; Ghader et al., 2024[17]). Other key determinants of adolescent mental well-being during the pandemic include educational restrictions, family conflicts and excessive social media exposure-all of which were substantiated in the thematic analysis of this review (Groarke et al., 2020[19]; Chavira et al., 2022[10]; Knowles et al., 2022[24]). Given the generalizability of these findings, it is evident that adolescents across the U.K. have been significantly impacted by these pandemic-related stressors.

Due to the global elevated unemployment rates associated with the pandemic and its restrictions, some parents lost their primary sources of income which in turn affected the household socioeconomic status (Tamirisa and Maringanti, 2024[37]). As a result, forced adaptation to the new financial situation in addition to the already ongoing restrictions was directly linked to mental instability in adolescents (Cohen et al., 2021[13]; Tamirisa and Maringanti, 2024[37]). This also increased family conflicts, further causing a ripple effect and leading to distress, anxiety, and other emotional challenges (Magson et al., 2021[26]).

Among the most critical emerging factors influencing adolescent mental health during the COVID-19 era is the availability of social support networks. Strong familial and peer relationships have been identified as key protective factors, mitigating the adverse effects of the pandemic on adolescent psychological well-being (Fledderjohann et al., 2021[16]). Human interaction is important in shaping and stabilizing mental health (Bell et al., 2023[6]). Hence, adverse interactions or its absence affects the wellbeing of adolescents, leading to loneliness and despondence. According to a similar review, with an increase in the length of time parents spent with their adolescents, the mental health status of the adolescents was significantly improved, suggesting a direct relationship between mental health and parental proximity (Tamirisa and Maringanti, 2024[37]). Conversely, social disconnection and relational instability have been linked to increased vulnerability to mental health disorders (Magson et al., 2021[26]).

Another interesting finding is the role of health provision, which has undergone significant transformations and the use of digital technologies, on the mental health outcomes of adolescents in the United Kingdom. The COVID-19 pandemic further highlighted these dynamics, thus necessitating a nuanced enquiry as they could have both ameliorating and exacerbating influences on adolescent mental health challenges (Bevan Jones et al., 2023[7]).

Additionally, a study by the University of Cambridge found that lack of computer access during the COVID-19 pandemic was associated with poorer mental health among young people, emphasizing the importance of addressing digital inequalities to safeguard mental well-being (University of Cambridge, 2022[40]). Contrastingly, The Guardian (Jenkins, 2024[22]) posits that excessive engagement with smartphones has been linked to the rapid deterioration of adolescent mental health, as evidenced by the Chennel 4 documentary called “Swiped”, which illuminated the severe impact of smartphone use on children's mental well-being, revealing heightened anxiety, stress, and even instances of self-harm associated with excessive phone use. Surprisingly, a temporary removal of phones resulted in a 17 % decrease in anxiety and depression symptoms, improved sleep, and better family interactions (Jenkins, 2024[22]). This is further buttressed by the increased records of cyberbullying due to the emerging habit of virtual engagements on social media and its effect on the typical adolescent such as the fear of missing out and the intrinsic need for external validation (Tamirisa and Maringanti, 2024[37]). Again, the higher rate of media engagement exposed the population to news of the pandemic which fueled anxiety in addition to existing emotional disturbances, especially as a result of misinformation (Vadivel et al., 2021[41]; Panchal et al., 2023[32]).

On the other hand, literature indicates the role of individual peculiarities in the response to the effects of the COVID-19 pandemic. While the mental condition of the majority of adolescents were facing a deterioration, there were reports of improved mental health in others (Raw et al., 2021[35]). A study in the USA attributed the experience of the latter category to healthy habits formed during the event (Raney et al., 2024[34]). More specifically, habits such as engagement in video games were listed as one of the escape methods used to cause a distraction in the minds of the adolescents (Cohen et al., 2021[13]). Some adolescents reported the positive use of the less social engagements for self-reflection and development which led to improved personalities (Bell et al., 2023[6]). Social media engagements were also leveraged to provide encouragement and boosters towards fulfilling endeavors such as meditation, exercise, skills building, and finding hobbies (Cohen et al., 2021[13]; Bell et al., 2023[6]).

One of the major recommendations rendered for mitigating the COVID-19 effect in adolescents is to find mental support (Bell et al., 2023[6]; Ghader et al., 2024[17]). This is because family warmth and support was significantly linked to improved mental health in adolescents by many researchers (Raw et al., 2021[35]; Vadivel et al., 2021[41]; Tamirisa and Maringanti, 2024[37]). The feeling of safety was statistically associated with parental presence, which promoted a positive outlook in adolescents from such household throughout the pandemic and in the post-pandemic era (Panchal et al., 2023[32]). On the other hand, family conflict was associated with behavioral and emotional symptoms, leading to the diagnosis of anxiety, depression, hyperactivity, and other psychological conditions (Magson et al., 2021[26]; Raw et al., 2021[35]). Hence, parents should be taught to manage their emotions during crisis to improve the relationships with their children and, consequently, make a positive impact on their mental and emotional wellbeing (Tamirisa and Maringanti, 2024[37]). 

In recent times, digital platforms have simplified access to mental health resources, offering interventions that are both scalable and cost-effective. For instance, MoodHwb, a program co-developed in Wales, provides adolescents with interactive components based on psychoeducation, cognitive-behavioral therapy, and interpersonal theory (Bevan Jones et al., 2023[7]). Also, the mental health text service, referred to as “Shout”, has engaged in over three million conversations since its inception in 2019, highlighting the potential of digital interventions in providing immediate assistance to those in distress (Perry, 2025[33]). 

Telemedicine should also be a tool in delivering psychological counseling and psychiatric diagnoses to encourage self-expression in these adolescents (Vadivel et al., 2021[41]). In addition, schools should provide counseling services as the majority of adolescents are associated with at least one educational institution and technology should be leveraged in reaching the members of this population for prompt intervention (Tamirisa and Maringanti, 2024[37]). As a region where the access to technology is recorded in the majority of its residents, there is a high feasibility of reaching the desired population via these methods as they present a cost-effective and fast intervention strategies (Bevan Jones et al., 2023[7]).

The mental health preparedness and action framework (MHPAF) was also recommended which is currently used in Kenya and the USA as a post-pandemic intervention method (Vadivel et al., 2021[41]). In addition, a combination of policy enactment and community enlightenment should be considered to reduce marginalization and cyberbullying (Vadivel et al., 2021[41]).

## Conclusion

This review provides a comprehensive synthesis of the mental health challenges experienced by adolescents in the U.K. in relation to the COVID-19 pandemic. The findings consistently indicate that depression and anxiety are the most prevalent mental health conditions within this population. Several key risk factors have been identified, including social restrictions, familial conflicts, gender disparities, financial instability, and the pervasive influence of social media. Given the significant and lasting implications of these findings, future adolescent mental health interventions should prioritize strategies aimed at mitigating these risk factors. The integration of digital technologies into health provision presents a complex interplay of benefits and detriments concerning adolescent mental health in the U.K. While these technologies offer innovative avenues for support and intervention, it is imperative to balance their use with strategies that mitigate potential harms. Policymakers, educators, and healthcare providers must collaborate to harness the advantages of digital health tools while implementing safeguards against their adverse effects, thereby promoting a holistic approach to adolescent mental well-being. By adopting targeted, evidence-based approaches that address these underlying determinants, policymakers and mental health professionals can foster more effective and sustainable improvements in adolescent psychological well-being.

## Declaration

### Conflict of interest

The authors declare that they have no conflict of interest.

### Authors' contribution

Conceptualization - Kelvin Kanayo Nwabueze, Chisom Chukwunonye, Kelechi Nelson Adindu

Methodology - Stella Chidinma Okolieze, Chinelo Grace Okengwu, Nnaemeka Akubue

Data collection - Ademola Onakoya, Nnaemeka Akubue, Kelvin Kanayo nwabueze

Data extraction - Ikponmwosa J. Otaniyen-Igbinoba, Kelvin Kanayo nwabueze, Ademola Onakoya

Review writing - Nnaemeka Akubue, Stella Chidinma Okolieze, Ikponmwosa J. Otaniyen-Igbinoba, Kelechi Nelson Adindu

Manuscript edit - Temiloluwa Ige, Kelechi Nelson Adindu

Manuscript review - Oluwaseyi Joy Alao, Chisom Chukwunonye.

### Artificial intelligence usage

The authors declare that no artificial intelligence tool was used throughout the conduction of this review. All research processes were completely performed by humans and all provided information are humanly generated.

## Figures and Tables

**Table 1:**
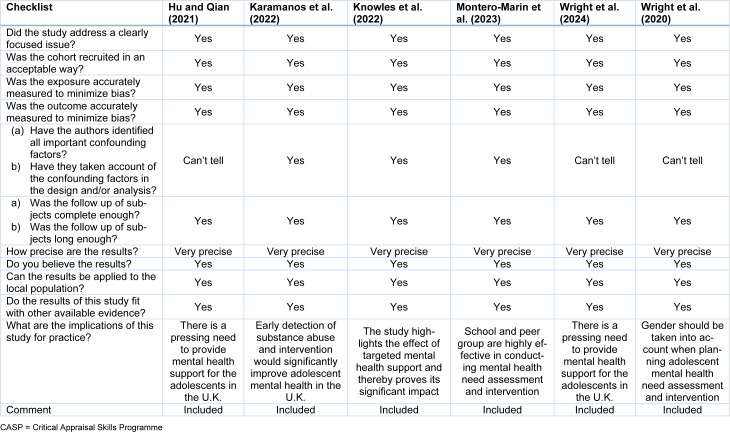
CASP tool for appraising cohort studies

**Table 2:**
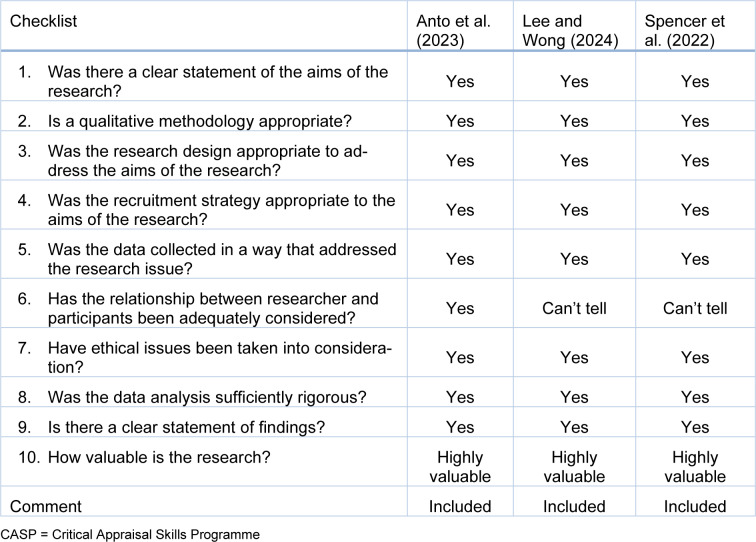
CASP tool for appraising qualitative studies

**Table 3:**
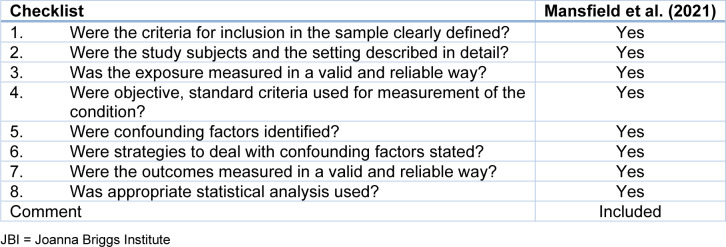
JBI tool for appraising cross-sectional studies

**Table 4:**
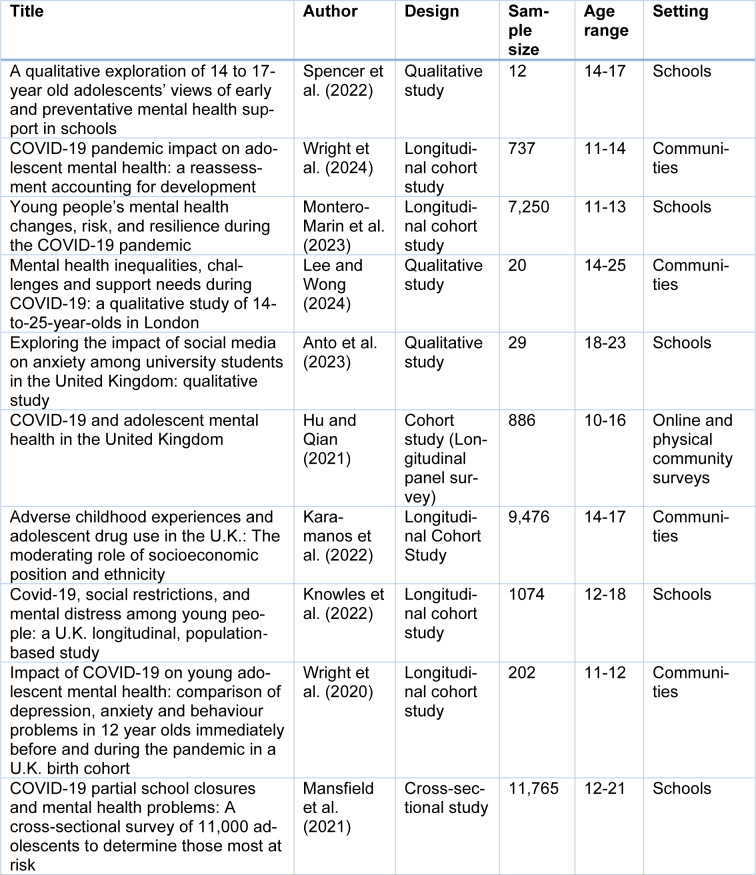
Summary of included studies
